# Development of a Region-Specific Physiologically Based Pharmacokinetic Brain Model to Assess Hippocampus and Frontal Cortex Pharmacokinetics

**DOI:** 10.3390/pharmaceutics10010014

**Published:** 2018-01-17

**Authors:** Zaril Zakaria, Raj Badhan

**Affiliations:** 1 Ministry of Health Malaysia, Block E1, E3, E6, E7 & E10, Parcel E, Federal Government Administration Centre, Putrajaya 62590, Malaysia; zakariz1@aston.ac.uk; 2 Applied Health Research Group, School of Life and Health Sciences, Aston University, Birmingham B4 7ET, UK; 3 Aston Pharmacy School, Aston University, Birmingham B4 7ET, UK

**Keywords:** PBPK, pharmacokinetics, CNS, brain, blood–brain barrier, microdialysis

## Abstract

Central nervous system drug discovery and development is hindered by the impermeable nature of the blood–brain barrier. Pharmacokinetic modeling can provide a novel approach to estimate CNS drug exposure; however, existing models do not predict temporal drug concentrations in distinct brain regions. A rat CNS physiologically based pharmacokinetic (PBPK) model was developed, incorporating brain compartments for the frontal cortex (FC), hippocampus (HC), “rest-of-brain” (ROB), and cerebrospinal fluid (CSF). Model predictions of FC and HC *C*_max_, *t*_max_ and AUC were within 2-fold of that reported for carbamazepine and phenytoin. The inclusion of a 30% coefficient of variation on regional brain tissue volumes, to assess the uncertainty of regional brain compartments volumes on predicted concentrations, resulted in a minimal level of sensitivity of model predictions. This model was subsequently extended to predict human brain morphine concentrations, and predicted a ROB *C*_max_ of 21.7 ± 6.41 ng/mL when compared to “better” (10.1 ng/mL) or “worse” (29.8 ng/mL) brain tissue regions with a FC *C*_max_ of 62.12 ± 17.32 ng/mL and a HC *C*_max_ of 182.2 ± 51.2 ng/mL. These results indicate that this simplified regional brain PBPK model is useful for forward prediction approaches in humans for estimating regional brain drug concentrations.

## 1. Introduction

Diseases of the central nervous system (CNS) present a considerable socioeconomic burden to healthcare systems, and are expected to exponentially increase with an ageing population. The World Health Organization (WHO) highlighted “brain diseases” as contributing to more than 35% of the total disease burden in Europe [1]. However, the majority of CNS disorders that warrant effective drug therapy currently lack examples of successful pharmacotherapy [2]. This failure is often related to the difficulty of finding medicines that can cross the blood–brain barrier (BBB) and enter the brain parenchyma [2]. The BBB plays a significant role in maintaining the neuroparenchymal microenvironment by protecting neural tissues from toxins [3]. Furthermore, the BBB presents an almost impermeable barrier to drug delivery for most small molecular weight compounds, thus often contributing to the attrition of many CNS drug development programs [2,4]. The ability to pragmatically assess the extent of CNS drug disposition in early discovery/development phases may potentially assist with understanding the characteristics of CNS uptake, and reduce the need for complex in vivo procedures to quantify CNS drug disposition. Traditional approaches to tackle this have often focused on determining the steady-state brain distribution of drugs in preclinical species, referred to as the brain-to-plasma ratio or *Kp_brain_*, and correlating this to the molecular properties in order to model/extrapolate brain distribution [5,6]. To account for the driving force for brain delivery (i.e., membrane permeability) and target site receptor occupancies, this is often corrected for the unbound brain-to-unbound plasma ratio (*Kp_uu_*_,*brain*_) [7,8].

There is, however, a significant lack of mechanistic predictive models capable of quantifying CNS drug disposition, particularly in different brain and CNS regions. In non-physiological empirical pharmacokinetic models, the CNS is described by either a one-compartment model (representing the brain) or a two-compartment model (representing brain interstitial fluid and brain intravascular fluid (IVF)), with such models often being used in conjunction with brain microdialysis data to describe CNS drug disposition [9,10]. Semi-physiological models have also been proposed in an attempt to describe drug disposition within the brain mechanistically [11,12,13,14,15,16,17,18,19].

However, all current semi-physiological and non-physiological models employed to describe CNS pharmacokinetics fail to consider regional CNS pharmacokinetics within district brain sections, which limits the application of such models to the assessment of regional brain extracellular fluid (ECF) drug disposition.

Recently, a series of publications by Yamamoto et al. [20,21,22,23] have established the basis for mechanistic regional pharmacokinetic modeling of CNS tissues; however, these models are based on a global regional model of the CNS (i.e., inclusion of regional CSF compartments), which would be more applicable to clinical sampling in humans (i.e., spinal CSF). Furthermore, such models were developed using population-based compartment modeling pharmacokinetics (e.g., NONMEM [24]). To address this limitation, PBPK can be used to mechanistically describe the drug concentration in tissues with consideration of regional drug brain tissue distribution [25,26]. A key benefit of the application of PBPK models is the ability to amalgamate existing relevant physiological processes, which may impact on the pharmacokinetics of compounds alongside a compound’s physicochemical properties to mechanistically describe a compound’s pharmacokinetics and allow both interspecies scaling and the prediction of whole organ and organ sub-compartment temporal concentration profiles. As opposed to empirical models, an integration between system-dependent (physiological) and compound-dependent parameters of PBPK models in predicting the compound’s PK profile has enabled an understanding of the underlying mechanisms of the PK [11,27] and recently been applied to model ECF pharmacokinetics of drugs [28,29,30].

The need for quantifying regional brain temporal concentrations is integral to expanding existing CNS PBPK modeling approaches, particularly for those drugs that are reported to be unevenly distributed within the brain [31,32]. The aim of this study is therefore to develop a PBPK model of the rat CNS that considers the whole brain ECF in addition to two regional compartments, namely the frontal cortex and hippocampus, to predict regional brain pharmacokinetics of phenytoin [31] and carbamazepine [33]. Furthermore, the model was expanded to predict the human regional brain pharmacokinetics of morphine.

## 2. Materials and Methods

A three-stage workflow methodology was applied to model development. Step 1 focused on the validation of a whole-body PBPK model incorporating a previously published CNS PBPK model [28], for the prediction of *Kp_uu,brain_* for 10 passively transported compound. Step 2 adapted this CNS PBPK model to include two regional brain compartments, namely the frontal cortex and hippocampus, and validated these against two reported studies of phenytoin [31] and carbamazepine [33] regional brain ECF temporal concentration from rodent microdialysis studies. Subsequently, Step 3 extrapolated the regional brain PBPK model to humans for the prediction of morphine pharmacokinetics based on reports of human brain microdialysis of morphine [34,35].

### 2.1. Step 1: A Whole-Body Physiologically Based Pharmacokinetic (PBPK) CNS Model

**Figure 1:**
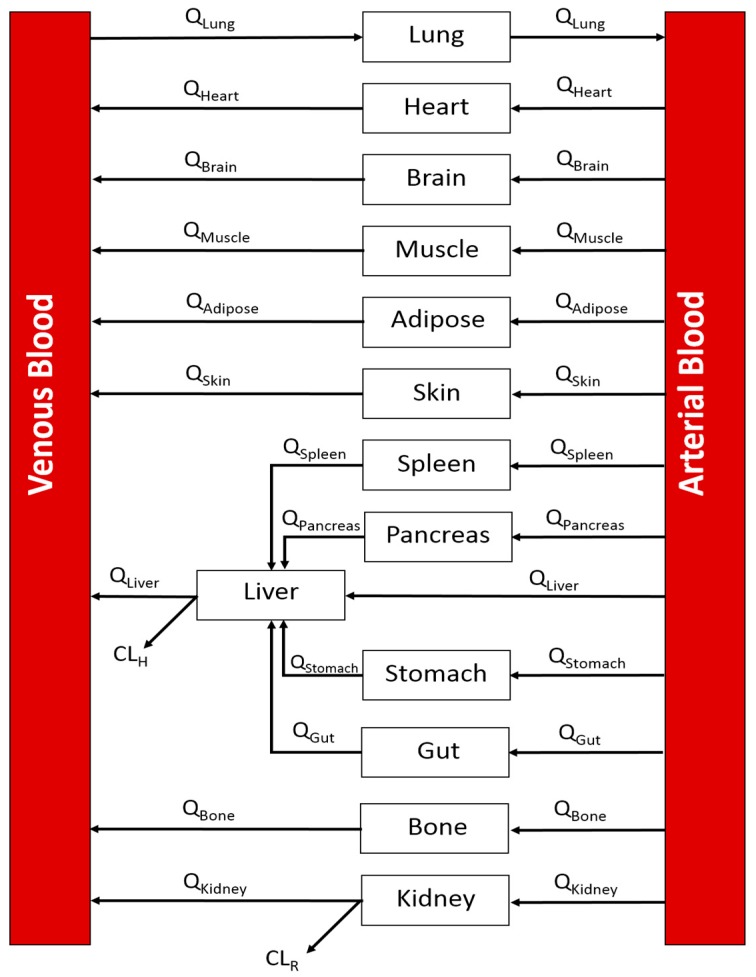
A generic whole-body PBPK model. Arrows indicated direction of blood flow. Q: blood flow; *CL_H_*: hepatic clearance; *CL_R_*: renal clearance.

A whole-body PBPK model was developed in MATLAB 9.1 (The MathWorks, Inc., Natick, MA, USA) [36]. The model incorporated the following compartments: lung, heart, brain, muscle, adipose, skin, spleen, liver, pancreas, gut, stomach, bone, kidney, arterial blood, and venous blood. All tissue compartments were modeled as well-stirred (Figure 1).

Ordinary differential equations were used to describe the whole-body PBPK model with all compartments, except CNS tissues, assumed as perfusion-limited/well-stirred and are fully described in the Supplementary Materials (Equation (S1)).

Drug removal from eliminating organs (liver and kidney) was described by either a hepatic clearance (*CL_H_*) or renal clearance (*CL_R_*) term. Hepatic clearance was derived from either in vitro intrinsic metabolic clearance (*CL_int_*_, *in vitro*_) or in vivo human blood or plasma clearance (*CL_b_* or *CL_p_*). Renal clearance was calculated using a GFR (glomerular filtration rate) correction approach [37].

Intrinsic clearance was scaled to a *CL_H_*, through the use of microsomal recovery (microsomal protein content: 45 mg protein/g liver) or hepatocellularity (130 × 10^6^ cells/g liver) and assuming a rat liver weight of 40 g/kg body weight [38,39,40], before being scaled using a well-stirred liver model (Equation (1)):

(1) $${\;\mathrm{CL}}_{H}=\frac{{\mathrm{fu}}_{p}\times {\mathrm{CL}}_{\mathrm{int},\;\mathrm{in}\;\mathrm{vivo}}\times Q_{L}}{Q_{L}+{\mathrm{fu}}_{p}\times {\mathrm{CL}}_{\mathrm{int},\;\mathrm{in}\;\mathrm{vivo}}/R_{b}}$$

Tissue volumes and blood flow rates were obtained from the published literature [11,41] (Table 1).

Ten passively transported compounds (benzylpenicillin, buspirone, caffeine, carbamazepine, diazepam, midazolam, phenytoin, sertraline, thiopental, and zolpidem) with reported unbound brain:unbound plasma partition coefficient (*Kp_uu,brain_*) were selected to validate the structure of the PBPK model. Physicochemical data for compounds are detailed in the Supplementary Materials (Section 1.1, Tables S1–S5). This approach required prediction of both plasma and brain concentration-time profiles to calculate the *Kp_brain_* (brain-to-plasma partition coefficient) (Equation (2)) and more specifically when corrected for the unbound fraction, *Kp_uu,brain_* (Equation (3)):

(2) $$Kp_{brain}=\frac{C_{brain}}{C_{plasma}}\;$$

(3) $$Kp_{uu,brain}=\frac{\int_{0}^{\infty }C_{u}brain\times dt}{\int_{0}^{\infty }C_{u}plasma\times dt}=\frac{AUC_{u,brain}}{AUC_{u,plasma}}$$

The brain was modeled with a perfusion-limited compartment (see Supplementary Materials Figure S1). Absorption (permeability clearances) from the BBB, protein binding (plasma, brain tissue and CSF), metabolic clearance and predicted tissue partition coefficients (*Kp_t_*) were previously collated by our group [28] and implemented within the model as described by Equation (S2) in the Supplementary Materials. In this approach, in vitro permeability was scaled to in vivo permeability through correction for the brain microvascular endothelial surface area (150 cm^2^·g·brain^−1^ for rats [42] or 157 cm^2^·g·brain^−1^ [43] for humans) and was parameterized into the appropriate unidirectional PS term (Equations (4) and (5)):

(4) $${\mathrm{PS}}_{blood-to-brain\;direction}={\mathrm{Papp}}_{\mathrm{AB}}\times \mathrm{Brain}\;\mathrm{weight}\times \mathrm{Surface}\;\mathrm{Area}\times \mathrm{CF}$$

(5) $$PS_{brain-to-blood\;direction}=Papp_{BA}\times Brain\;weight\times Surface\;Area\times CF$$

where brain weight was assumed to be 1.8 g in rats, 0.36 g in mice, and 1500 g in humans [44,45,46].

The CF term relates to an in vitro to in vivo extrapolation factor that corrects for the absent physiological conditions inherent in the determination of the in vitro permeability [29,30]. It is also important to note that, for actively transported compounds, CF can be replaced by a relative expression factor (REF) that accounts for the differences in transporter abundances from the in vitro system to the in vivo species being studied [29,30]. CF was assumed to be “1” in the absence of any parameter estimation approaches. When only a single Papp was reported in the literature, the resultant predicted PS was assumed to be bidirectional. Furthermore, for active efflux compounds, the PS*_blood_*_-*to*-*brain*_ was assumed to be bidirectional and the active efflux component was applied through correction of the PS*_brain_*_-*to*-*blood*_ of the efflux ratio of the substrate [28].

All compounds were simulated using IV bolus doses.

The validity of individual compounds was assessed using a fold-error (*FE*) method whereby whenever the observed *Kp_uu,brain_* values were determined to be more than the predicted *Kp_uu,brain_* values,

(6) $$FE=\frac{Kp_{uu,brain}Observed}{Kp_{uu,brain}Predicted}$$

If however, the predicted *Kp_uu,brain_* values were more than the observed *Kp_uu,brain_* values,

(7) $$FE=\frac{Kp_{uu,brain}Predicted}{Kp_{uu,brain}Observed}$$

### 2.2. Step 2: Development of a Rat Regional Brain PBPK Sub-Model

A study by Walker et al. (1996) [31] reported the regional brain concentration of phenytoin in distinct brain regions of the rat, namely the hippocampus and frontal cortex. A further study by Van Belle et al. (1995) [33] also reported carbamazepine regional brain concentrations in the hippocampus. These studies were used to validate the regional brain PBPK sub-model. Compound-specific parameters for phenytoin and carbamazepine, along with permeability clearances across the hippocampus, frontal cortex, and the rest of the brain tissues were obtained from previously collated in vitro permeability data [28].

**Figure 2:**
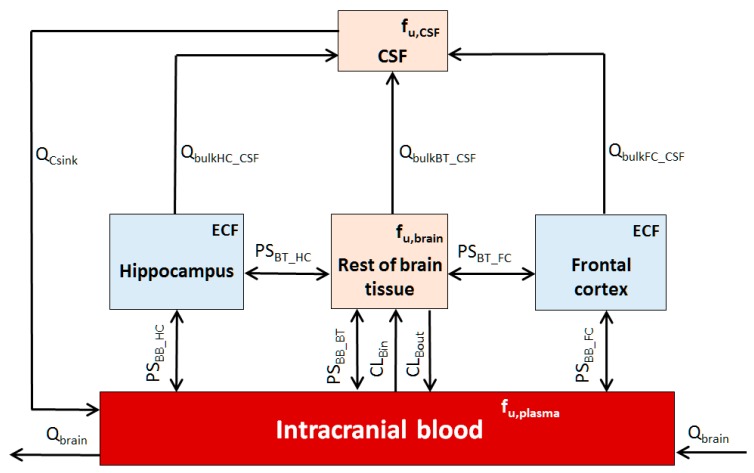
Five-compartmental rat CNS PBPK brain model. Q: blood flow; PS: permeability surface-area; BB: intracranial blood; HC: hippocampus; FC: frontal cortex; C and CSF: cerebrospinal fluid; BT: brain tissue; f_u_: drug fraction unbound in brain regions (*fu_,brain_*), CSF (*fu_,CSF_*) or plasma (*fu_,plasma_*).

Model development adapted a previously reported CNS PBPK model [28] to include a hippocampus and frontal cortex compartment (Figure 2) and was applied to the whole-body PBPK model, with systems parameters detailed in Table 2.

In the development of this model, the following assumptions are made:

1. The CNS is represented by five compartments, namely CSF, intracranial blood, rest of brain tissue, frontal cortex, and hippocampus;
2. All compartments are well stirred, with permeability barriers between the intracranial blood and brain;
3. There is no rate-limiting diffusion barrier between the ECF and CSF, and the drug equilibration between these two compartments is rapid [29];
4. Only an unbound drug, governed by unbound fraction in plasma (*fu,_plasma_*), brain tissue (*fu,_brain_*) or CSF (*fu,_CSF_*), was considered capable of crossing permeability barriers;
5. In the absence of published regional *fu,_brain_*, the unbound brain fraction was assumed to be equivalent for all brain regions (i.e., hippocampus, rest of brain, and frontal cortex) [47];
6. Within the extracellular space of the brain, fluids move either by diffusion or by bulk flow (*Q_bulk_*) [48];
7. Where absent from the literature, hippocampus and frontal cortex volumes scaled from mice to rats based on brain weight ratio-scalars [28,45,46,49];
8. Due to the absence of regional brain in vitro or in vivo permeability data, the regional brain bi-directional passive transport (*PS*) term was scaled from in vitro Papp and corrected for the regional tissue weight (Table 2, assuming density = 1) using Equations (4) and (5), wherein the term “brain weight” is replaced by “regional brain weight”;
9. The temporal concentration profile of the drug in the regional brain ECF would mimic the biophase sampled during microdialysis studies [50];
10. Since the liver was considered the only site of clearance for phenytoin based on the literature [51], the prediction for unbound renal clearance (*CL_R_*) was excluded from the simulation;
11. Active transport from brain tissues (Efflux: *CL_Bout_*; Influx: *CL_Bin_*) can be determined as described in our previous CNS PBPK model [28].

The CNS PBPK model equations are detailed in the Supplementary Materials (Section 2, Equations (S3)–(S6)), with compound physicochemical data detailed in the Supplementary Materials (Section 2.1, Table S6).

For the rat brain PBPK model, the tissue volumes and blood flow rates were obtained from the published literature (Table 2). Subsequently, the five-compartment brain model was applied to predict plasma, rest of brain, hippocampus, and frontal cortex concentration profiles following an intraperitoneal dose of 50 mg/kg of phenytoin [31] or a 10-mg IV infusion (10 min) of carbamazepine.

In order to account for the uncertainty in the ECF volumes of regional brain compartment, Monte Carlo simulations were used to incorporate a 30% CV (log-normal distributed) on the fixed estimates of ECF compartment volumes (simultaneous applied and simulated to the rest of brain, hippocampus and frontal cortex) resulting in at least 3000 runs per compound (1000-per compartment). This was applied using simulations for both rat (Step 2) and human (Step 3) models. The resultant 5th and 95th percentiles were graphically assessed.

To assess the impact of PS parameter uncertainty on model predictions, a sensitivity analysis was conducted to assess the impact of variation in PS_HC_BT_ and PS_BB_HC_ and PS_FC_BT_ and PS_BB_FC_, on the hippocampus and frontal cortex *C*_max_ over a PS range of 0.01 to 100 mL/min using phenytoin as a model compound. Three-dimensional mesh plots were used to assess this relationship graphically.

### 2.3. Step 3: Development of a Human Regional Brain PBPK Sub-Model

To explore the possibility of utilizing the regional brain PBPK model to predict human brain pharmacokinetics, human CNS physiological parameters were used to develop a human regional CNS PBPK model (Table 2) based upon the regional brain model described in Section 2.2. Despite limited human brain concentration data being reported in the literature, two studies were chosen that reported morphine brain concentrations in patients who suffered from traumatic brain injury, acquired using microdialysis cerebral catheter insertion in “better” or “worse” brain tissues, as determined by computed tomography scanning [34,35]. Systems parameters for the human CNS PBPK model are detailed in Table 2 and morphine-specific parameters are detailed in the Supplementary Materials (Section 2.1, Table S6).

## 3. Results

### 3.1. Step 1: Validation of the PBPK Model

**Figure 3:**
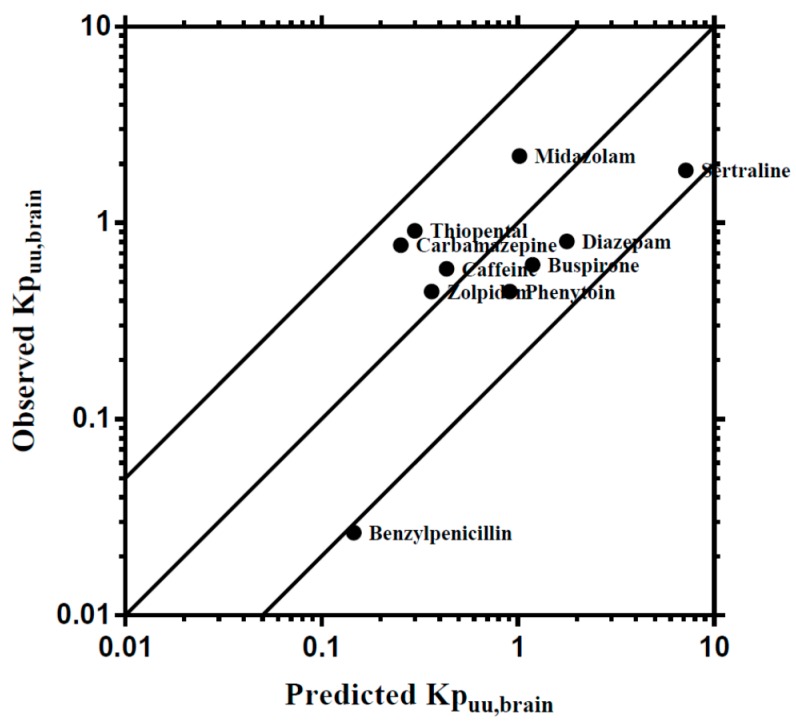
Comparisons of predicted and reported *Kp_uu,brain_* in rat. The solid bold mid-line represents the line of unity, and solid outer lines represent 5-fold prediction error.

To develop a broader regional CNS PBPK model, this step focused upon the development of a base PBPK model consisting of a whole-body PBPK incorporating a simplistic 1-compartment model of the brain. Predictions of brain temporal concentration profiles were surmised using the unbound brain: plasma ratio (*Kp_uu,brain_*), which is widely used to assessed brain drug partitioning. Validation of the WB-PBPK examined the ability of the model to predict *Kp_uu,brain_* in rats for 10 compounds demonstrating passive absorption across the BBB that were previously used in PBPK modeling by our group [28]. The WB-PBPK model was capable of predicting *Kp_uu,brain_* to within 5-fold of the reported *Kp_uu,brain_* for all compounds except benzylpenicillin, which was 5.34-fold over predicted (Figure 3).

### 3.2. Step 2: Development of a Rat Regional Brain PBPK Sub-Model

#### 3.2.1. Case 1: Phenyotin

The base PBPK model described in Step 1 was adapted to replace the one-compartment brain model with a five-compartment regional brain model. This model was then used to predict phenytoin plasma and regional brain concentrations.

**Figure 4:**
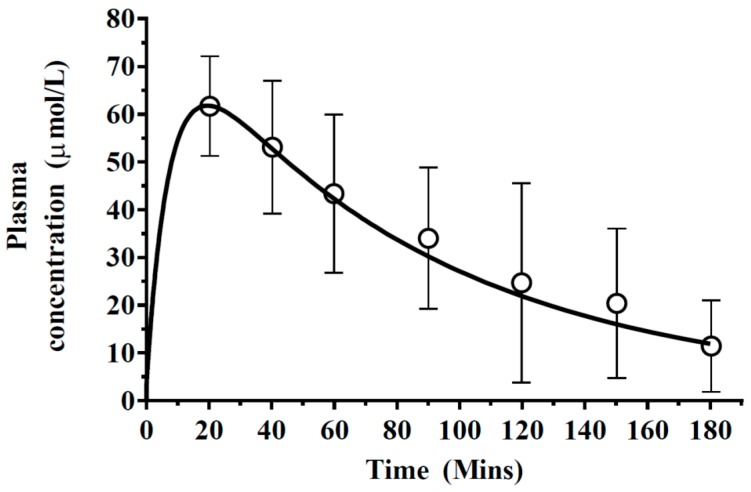
Simulated mean phenytoin plasma concentration–time profiles. A 50 mg/kg phenytoin dose was simulated (solid line) with reported literature data, represented by open circles with error bars representing standard deviations.

Predictions of phenytoin plasma concentration profiles were subsequently simulated and found to be within the range of observed profiles (Figure 4), with a predicted *C*_max_ (61.79 µmol/L) similar to that reported by Walker et al. (1996) [31], 61.69 ± 4.7 µmol/L. Furthermore, a similar *t*_max_ was predicted compared to that reported by Walker et al. [31], approximately 20 min (Figure 4).

**Figure 5:**
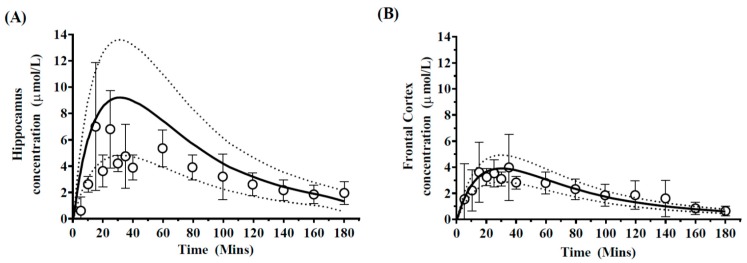
Simulated mean phenytoin hippocampus and frontal cortex concentration–time profiles. Simulated mean values of phenytoin-time profiles in (**A**) hippocampus and (**B**) frontal cortex after a 50 mg/kg dose of phenytoin. Open circles and errors bars represent literature reported mean and ±SD in 5 rats. The solid black line represents model prediction mean profiles and dashed lines indicated 95th and 5th percentiles.

Prediction of regional brain concentrations was accomplished through application of the five-compartment brain model, which incorporated distinct hippocampus and frontal cortex compartments. When accounting for uncertainly in model parameter predictions, model predictions were compared to those reported using microdialysis sampling in the hippocampus and frontal cortex, as reported by Walker et al. (1996) [31] and were generally in agreement with observed profiles in each brain region (Figure 5).

Model predicted *C*_max_ and AUC were within 2-fold of that reported [31] (Table 3). Predictions of hippocampus *t*_max,_ approximately 20 min, were slightly over-predicted compared to the observed *t*_max_ of 15 min. For the frontal cortex mean concentration, *C*_max_ was predicted at 3.87 ± 0.24 µmol/L and was consistent with the published literature *C*_max_ of 3.98 ± 1.1 µmol/L (Figure 5B).

In both cases, the *afe* and *rmse* of 0.92 and 0.40 respectively, were indicative of good model prediction. Furthermore, predictions of the regional *Kp_uu,brain_* for the hippocampus (0.12) and frontal cortex (0.057) were within 2-fold of the reported regional *Kp_uu,brain_* of 0.11 for the hippocampus and 0.08 for the frontal cortex.

#### 3.2.2. Case 2: Carbamazepine

**Figure 6:**
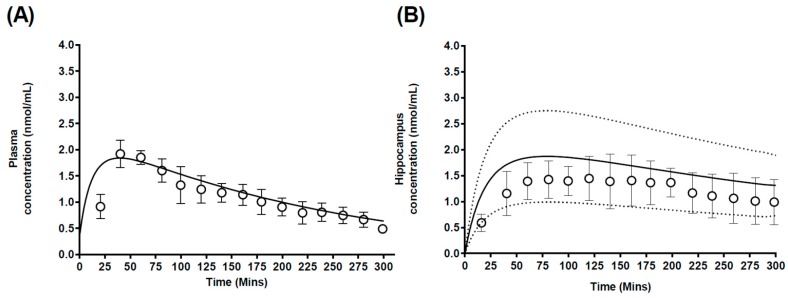
Simulated mean carbamazepine plasma and hippocampus concentration-time profiles. Simulated mean values of carbamazepine-time profiles in (**A**) plasma and (**B**) hippocampus after a 2.5 mg/kg carbamazepine dose. Open circles and errors bars represent literature reported mean and ±SD. Solid black line represents model prediction mean profiles and dashed lines indicated 95th and 5th percentiles.

Predictions of carbamazepine plasma concentration profiles were found to be within the range of the observed data (Figure 6A), with a predicted *C*_max_ (1.81 nmol/mL) similar to that reported by Van Belle et al. (1996) [33], 2.14 ± 0.27 nmol/mL (Table 4). Furthermore, a similar *t*_max_ was predicted, 39 min, compared to the reported *t*_max_ [33] of approximately 44 ± 9 min (Table 4).

Van Belle et al. [33] reported carbamazepine hippocampus pharmacokinetics following a single dose to rats and this was used as a basis to further validate the regional brain PBPK model. The model predicted plasma (Figure 6A) and hippocampus (Figure 6B) *C*_max_ and AUC to within 2-fold of the reported values (Table 4). Furthermore, predicted regional *Kp_uu,brain_* were within 2-fold of the reported *Kp_uu,brain_* (reconstructed from the AUC ratios) [33], 0.79 and 1.02 respectively.

#### 3.2.3. Model Sensitivity Analysis

**Figure 7:**
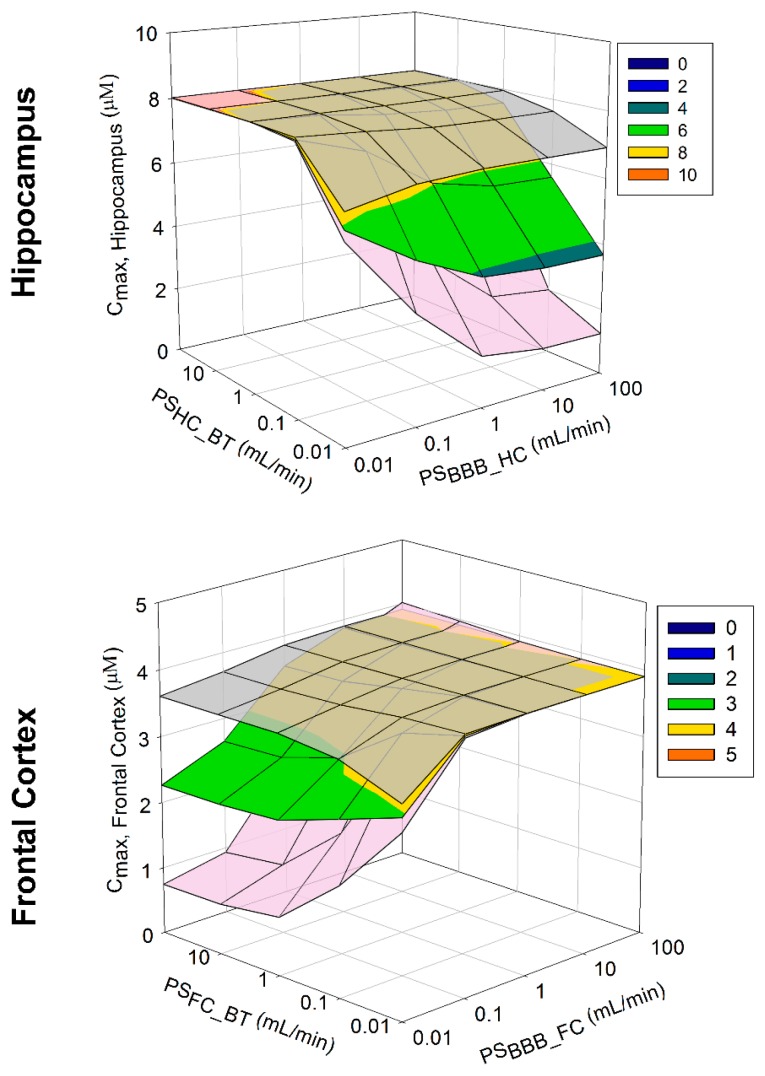
Model sensitivity analysis of brain PS on *C*_max._ Sensitivity analysis of the impact of variation in PS on the hippocampus (upper panel) or frontal cortex (lower panel) phenytoin *C*_max_. Gray mesh indicates profiles where PS_BBB_BT_ is 10-fold lower and pink mesh indicates profiles where PS_BBB_BT_ is 10-fold higher than that presented in the associated multicolor mesh plots. PS: permeability surface area product; HC_BT (hippocampus and brain tissue); BBB_HC (cerebral microvasculature [blood brain barrier] and hippocampus); FC_BT (frontal cortex and brain tissue) and BBB_FC (cerebral microvasculature [blood brain barrier] and frontal cortex).

To assess the impact of parameter uncertainty on model predictions, a sensitivity analysis assessed the impact of variation in PS_HC_BT_, PS_BB_HC_, PS_FC_BT_ and PS_BB_FC_ on phenytoin (as a model compound) hippocampus and frontal cortex *C*_max_ over a PS range of 0.01 to 100 mL/min (Figure 7). Model predictions were generally sensitive to changes in both drug flux into each compartment (PS_BBB_HC_ or PS_BBB_FC_) and out of each compartment (PS_HC_BT_ or PS_FC_BT_). Irrespective of changes in hippocampus PS over the range simulated, predicted *C*_max_ spanned 3.7 to 8 µM. Furthermore, variations in frontal cortex PS resulted in a predicted *C*_max_ spanned 2.3 to 3.9 µM. Assuming regional differences in the HC and FC compared to the rest of the brain, where flux across the regional BBB located at the “rest of brain” was ten-folder greater than that of the HC or FC, limited sensitivity was simulated across any change in PS_BBB_HC_, PS_BBB_FC_, PS_HC_BT_ or PS_FC_BT_.

### 3.3. Step 3: Development of a Human Regional Brain PBPK Sub-Model

In an attempt to predict regional brain concentrations in humans, we utilized data reporting morphine brain concentrations in patients who suffered from traumatic brain injury using microdialysis cerebral catheter insertion in “better” or “worse” brain tissues, as determined by computed tomography scanning [34,35].

**Figure 8:**
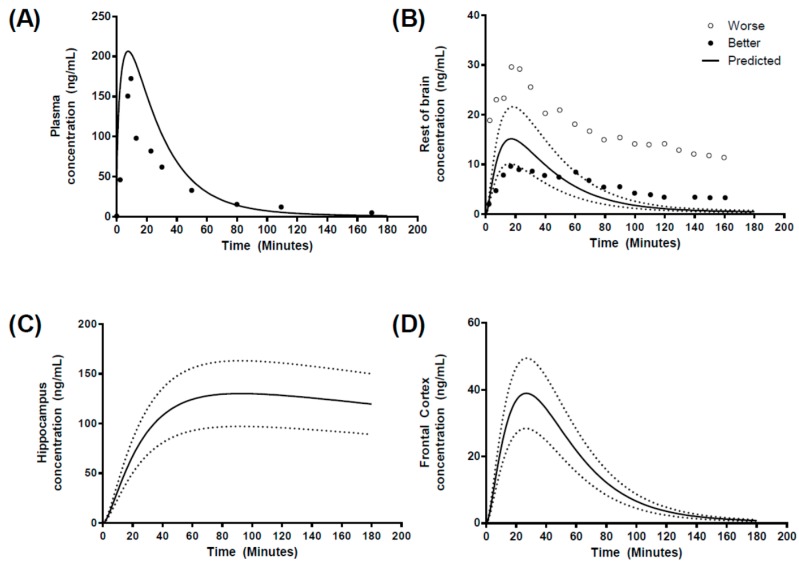
Simulated human morphine concentration-time profiles. Predicted concentration-time profiles for (**A**) plasma, (**B**) rest of brain, (**C**) hippocampus and (**D**) frontal cortex, following a 10 mg IV-infusion over 10 min. Circles represent literature reported values. Solid black line represents model prediction mean profiles and dashed lines indicated 5th and 9th percentiles. “Better” and “worse” regional brain morphine concentrations are highlighted by solid or open circles in (**B**).

The plasma concentration–time profile was well predicted (Figure 8A) with *C*_max_, *t*_max_ and AUC all within 2-fold of the reported values (Table 5). In the absence of human hippocampus or frontal cortex temporal concentration profiles, we compared the reported profiles for “better” and “worse” brain morphine temporal concentration profiles to those generated within the “rest of brain” compartment within the regional brain PBPK model (Figure 8B). The model predicted a ROB *C*_max_ of 14.5 ± 4.21 ng/mL, which was within the range reported for both “better” and “worse” brain tissue, in addition to calculated AUC beings within 2-fold of those reported (Table 5). However, *t*_max_ was 2.5-fold underpredicted. For regional brain compartments, the hippocampus exhibited a slow transfer of morphine leading to a *t*_max_ of 79.6 min and *C*_max_ of 124.4 ± 41.2 ng/mL, while the frontal cortex *t*_max_ was shorter (26.5 min) with a *C*_max_ of 38.91 ± 15.78 ng/mL (Figure 8C,D).

## 4. Discussion

Central nervous system (CNS) disorders affect millions of people worldwide despite the availability of a wide range of established treatments [1]. The primary challenge to CNS drug delivery is the penetration of the blood–brain barrier in order to attain a sufficiently high biophase concentration for a clinical effect. Given the lengthy discovery and development times associated with CNS drug development, the application of mechanistic pharmacokinetic modeling has emerged to bridge the gaps between in vivo and in vitro approaches to expedite extrapolation of the pharmacokinetics of drug compounds and to aid in the selection of appropriate doses for clinical studies [63,64].

The primary aim of this research was to employ mechanistic pharmacokinetic modeling approaches to develop models capable of conducting robust in vitro to in vivo correlation and thus allow interspecies extrapolations (rodent to human). Such approaches are based around a mechanistic set of physiological (“systems”) parameters describing the physiology of the model system (e.g., rodents or humans) and in vitro derived or estimated drug (“compound”) parameters.

Such extrapolations will enable the quantification and prediction of the extent of drug delivery to the brain and wider CNS across drug barrier sites, namely, the BBB and the regional brain area. These mechanistic platforms are in line with a replacement, reduction and refinement concept that is integrated into the drug discovery framework [65]. The aim of this study was therefore to develop a PBPK model of the rat CNS that considered the whole brain ECF in addition to the frontal cortex and hippocampus, to predict regional brain pharmacokinetics of phenytoin and carbamazepine in rats, in addition to the prediction of human regional brain pharmacokinetics or morphine.

### 4.1. Validation of the PBPK Model

To develop an accurate brain PBPK model that can predict human drug concentrations from a limited set of routinely available pre-clinical and in vitro drug-specific parameters, a robust validation process is essential to determine the prediction accuracy and precision. A rat CNS PBPK model developed by Ball et al. (2012) [30] was selected to confirm successful base model development. Initial validation was conducted by comparing the *Kp_uu,brain_* values between the predicted and published data for passively transported compounds, namely benzylpenicillin, buspirone, caffeine, carbamazepine, diazepam, midazolam, phenytoin, sertraline, thiopental, and zolpidem [28]. Model predictions were all within 5-fold of the observed *Kp_uu,brain_*, with prediction of *Kp_uu,brain_* for benzylpenicillin being 5.5-fold overpredicted (Figure 3). This overprediction found in benzylpenicillin may be a result of the involvement of unclarified molecular active transport mechanism through the BBB, as reported by Suzuki et al. [66,67], where the rapid CNS elimination was not captured during the simulation.

As the description of the brain compartment using a simplistic permeability limited compartment is not physiologically relevant, it would be expected that model predictions of temporal brain concentrations would, therefore, be less accurate and this would account for the large error range simulated. This basic CNS PBPK model was subsequently adapted and built upon in Step 2 to propose a regional brain CNS PBPK model that was more mechanistically derived.

### 4.2. Prediction of Regional Brain Concentrations in Rats

In order to expand upon this previously developed model, we adapted the basic CNS PBPK model to include two further tissue compartments, namely the frontal cortex and hippocampus. In this process, we identified two candidate compounds to validate our adapted model against, phenytoin and carbamazepine. Both compounds have been administered to rats and region-specific brain microdialysis conducted to assess the CNS pharmacokinetics. Frontal cortex and hippocampus phenytoin concentrations had been previously reported by Walker et al. (1996) [31], with Van Belle et al. (1995) [33] also reporting carbamazepine regional brain concentration in the hippocampus. The PBPK model incorporated an in situ permeability surface area (PS) previously reported in rodents to drive diffusion from the plasma circulation into the CNS. The resultant predictions of plasma and regional concentrations were within the range of concentrations reported for both compounds (Figure 4, Figure 5 and Figure 6), with the majority of model predictions pharmacokinetic parameters within 2-fold of that observed (Table 4 and Table 5).

#### Model Sensitivity Analysis

Monte-Carlo based model sensitivity analysis was first addressed by assessing the uncertainty in our calculation of regional brain compartments volumes on predictions of regional brain concentrations. The resulting prediction range (5th–95th percentiles) adequately spanned a similar range to the reported range in the observed datasets from both Walker et al. (1996) [31] (Figure 5) and Van Belle et al. (1995) [33] (Figure 6) and highlights the importance of the potential inter-individual variability in regional brain tissue volume on overall model predictions.

Assuming the permeation of drug across the brain microvascular is uniform (i.e., no regional differences), variations in the inter-regional brain permeability (PS_HC_BT_ or PS_FC_BT_) of the drug would play a minimal role in influencing regional brain C_max_. Furthermore, assuming that the regional brain penetration of drug was non-uniform across the brain, a 10-fold lower or 10-fold higher shift in PS_BBB_BT_ would significantly increase (10-fold lower) or reduce (10-fold higher) overall regional brain C_max_ (Figure 7).

### 4.3. Prediction of Regional Brain Concentrations in Humans

The prediction of human CNS pharmacokinetics, from preclinical data, would provide an invaluable approach to assessing the usefulness of candidate molecules progressing through the drug development process.

Human brain pharmacokinetics data is extremely sparse in the literature; a study was selected that applied microdialysis to quantify morphine pharmacokinetics in human brain tissue, where a relatively rich brain pharmacokinetic profile was available. These data were available for “brain tissue”, and we assumed this was equivalent to the “rest of brain” compartment within our five-compartment brain model. The resultant model predictions resulted in a reasonable prediction of the shape of the concentration profiles along with a good estimate of the *C*_max_ and AUC, the former of which was predicted within the “range” of “better” and “worse” *C*_max_ reported in the observed datasets (Figure 8). However, the prediction of the terminal elimination phase was poorer than expected, although the reported data only illustrated data points for two representative patients, the distribution of resultant morphine concentrations at each time point was not reported and hence we were unable to ascertain the intra-individual variability.

As a first principles approach, we have been able to capture the pharmacokinetics of morphine in human brain tissue and the validated “rest of brain” compartment. Assuming PS is scaled from in vitro Papp based on correction for surface area (cm^2^/g tissue), the PS would be “corrected” for overall surface area based on the gross tissue weight. Furthermore, the small regional mass of the hippocampus would result in a highly localized concentration of morphine, which would slowly diffuse out of the brain tissue as a result of the smaller surface area. Similarly, regional differences in both morphine [68] and biperiden have been reported in rat brains and biperiden [69].

Further although this model did not consider active transport substrates, it would be possible to model the active transport of, for example P-glycoprotein substrates. This is made possible by the availability of absolute protein abundance data for a range of transporter proteins at brain barriers as a result of the application of quantitative proteomics [70,71]. Furthermore, we have previously demonstrated the application of this approach to the prediction of *Kp_uubrain_* (for whole brain) for 11 active transporter substrates using a similar CNS PBPK [28], where an active transport permeability surface area (PS) can be determined by the use of a corrected efflux ratio (to account for the differentiation between purely active and purely passive transport) [72,73] in addition to accounting for the abundance of the transporter protein in question factor [28].

Finally, key to driving regional brain drug concentration predictions would be accounting for any potential regional differences in non-specific brain tissue binding (i.e., a brain regional specific fu_bt_). In the absence of any reported regional brain *fu_bt_* data, we assumed *fu_bt_* was uniform across all brain regions. Any regional differences in grey/white matter phospholipid /lipid content may result in localized differences in *fu_bt_*. Indeed, it has been reported that differences in lipid content do exist when comparing white and grey matter regions [74,75,76,77]. Given these potential regional differences in brain composition, the application of techniques such as equilibrium dialysis should be encouraged to further investigate and determine fu_bt_ for specific brain regions to provide more appropriate input data in the model.

## 5. Conclusions

A regional brain PBPK model was developed for rats and extended to model the human regional brain pharmacokinetics of morphine. While the limiting factor in the application of this model to human CNS pharmacokinetics is the paucity in human brain (whole) or regional brain drug concentrations, with the greater application of cranial microdialysis it would be possible to further refine the proposed model for application in regional brain concentration. Nonetheless, the manuscript has successfully proposed a simplified first principles approach to the development of a regional brain CNS PBPK model.

## Figures and Tables

**Table 1:** System-related parameters used for the rat whole-body PBPK model.

Tissue	Perfusion		Volume	
	Rat	Human	Rat	Human
	(mL/min)	(mL/min)	(mL)	(mL)
Adipose	4.72	277.5	19.03	10,725
Bone	8.08	270	10.37	9300
Brain	1.12	750	1.43	1552.5
Gut	12	975	6.75	1770
Heart	3.2	160.5	0.825	285
Kidney	11.6	1177.5	1.825	330
Liver	20	1575	10.3	1807.5
Lungs	80	5325	1.25	1252.5
Muscle	18.96	802.5	101	32,175
Pancreas	1	142.5	1.3	90
Skin	4.08	322.5	47.5	8325
Spleen	0.88	82.5	0.5	202.5
Arterial blood	-	-	6.8	1927.5
Venous blood	-	-	13.6	3855

**Table 2:** System-related parameters used for the brain PBPK model.

	Rat	Human
Flow Rates ^a^	Q (mL/min)	
Rest of brain tissue to CSF (bulk flow)	0.00024	0.285
Hippocampus to CSF (bulk flow)	0.00002	0.00114
Frontal cortex to CSF (bulk flow)	0.00005	0.0566
CSF production rate	0.0037 ^b^	0.35 ^c^
CSF absorption (Qc_sink_) ^d^	0.0037	0.35
Volume	V (mL)	
Intercranial blood ^e^	0.025	75
Rest of brain tissue ^f^	1.222	1211
* Rest of brain tissue ECF ^e^	0.243	267
Hippocampus	0.093 ^g^	5.68 ^h^
* Hippocampus ECF ^e^	0.019	1.07
Frontal cortex	0.233 ^i^	283 ^j^
* Frontal cortex ECF ^e^	0.038	53.2
CSF	0.25 ^k^	160 ^l^

* Monte Carlo simulations were applied to address uncertainty in true parameter value. A 30% CV was applied as the boundary conditions and predictions conducted with all parameters identified simultaneously using a log-normal distribution with at least 3000 iterations per compound. ^a^ Regional brain ISF bulk flow was assumed to be 0.2 µL/min·g brain [52] and assumed to be species independent; ^b^ Taken from Harnish et al. [53]; ^c^ Taken from Brinker et al. [54]; ^d^ Assuming that the rate of CSF absorption is the same with CSF production rate [55]; ^e^ Calculated by assuming fractional volume of brain intravascular fluid is 0.014 and fractional volume of brain interstitial space 0.188 [56]; ^f^ Assumes average brain weight of 1.8 g in rats, 0.36 g in mice and 1500 g in humans [44,45,46]; ^g^ Taken from Lee et al. [57]; ^h^ Taken as mean of total hippocampal volume (right and left) [58]; ^i^ Scaled based on a mean mouse frontal cortex volume of 0.0467 mL [59] and a scalar of 5 (ratio of rat brain weight:mouse brain weight) or 4166 (ratio of human brain weight:mouse brain weight); ^j^ Taken as mean of range reported values from Semendeferi et al. [60]; ^k^ Taken from Bass and Lundbord [61]; ^l^ Taken from Sakka et al. [62].

**Table 3:** Summary of predicted and observed pharmacokinetic parameters of phenytoin in plasma, hippocampus and frontal cortex in rats.

	Plasma		Hippocampus		Frontal Cortex	
	*C*_max_	AUC	*C*_max_	AUC	*C*_max_	AUC
	(µmol/L)	(µmol/L·min)	(µmol/L)	(µmol/L·min)	(µmol/L)	(µmol/L·min)
Predicted	61.79	5891.97	8.62 ± 3.42	718.29 ± 18.31	3.87 ± 0.24	340.47 ± 11.53
Observed	61.69 ± 4.7	5924.55 ± 340.4	7.00 ± 2.2	594.74 ± 21.2	3.98 ± 1.1	370.97 ± 17.1

AUC is calculated as AUC_(0-last)_; Data represents mean ± SEM.

**Table 4:** Summary of predicted and observed pharmacokinetic parameters of carbamazepine in plasma and hippocampus brain regions in rats.

	Plasma		Hippocampus		Frontal Cortex	
	*C*_max_	AUC	*C*_max_	AUC	*C*_max_	AUC
	(µmol/L)	(µmol/L·min)	(µmol/L)	(µmol/L·min)	(µmol/L)	(µmol/L·min)
Predicted	61.79	5891.97	8.62 ± 3.42	718.29 ± 18.31	3.87 ± 0.24	340.47 ± 11.53
Observed	61.69 ± 4.7	5924.55 ± 340.4	7.00 ± 2.2	594.74 ± 21.2	3.98 ± 1.1	370.97 ± 17.1

Data represents mean ± SD.

**Table 5:** Summary of predicted and observed pharmacokinetic parameters of morphine in plasma and regional brain compartments in humans.

Compartment		*C*_max_	AUC	*t*_max_
		(ng/mL)	(ng/mL·min)	(min)
Plasma	Predicted	208.2	5363	7.2
	Observed	178	7513 ± 124	9.8
Better Brain	Observed	10.1	941.7	31.4 ± 17.1
Worse Brain	Observed	29.8	2732	17.8 ± 2.3
Rest of brain	Predicted	14.5 ± 4.21	815 ± 93	18.1
Hippocampus	Predicted	124.4 ± 41.2	19,971 ± 3791	79.6
Frontal Cortex	Predicted	38.9 ± 15.7	2444 ± 153	26.5

Data represents mean ± SD.

## Supplementary Materials

Supplementary materials can be found online at the following link www.mdpi.com/1999-4923/10/1/14/s1. The regional brain PBPK model has been provided as a ‘systems-biology markup language’ (SBML) file in an extended markup language format (.xml) format.

Supplementary File 1
