# Supplementary material for: Development of a Region-Specific Physiologically Based Pharmacokinetic Brain Model to Assess Hippocampus and Frontal Cortex Pharmacokinetics
Source: Pharmaceutics. 2018 Jan 17;10(1):14. doi: 10.3390/pharmaceutics10010014 (PMC5874827; doi:10.3390/pharmaceutics10010014)
Supplement: Supplementary File 1 [file pharmaceutics-10-00014-s001.zip › pharmaceutics-263994-supplementary.pdf]

Supplementary materials: Development of a Region-Specific Brain Physiologically-Based Pharmacokinetic Model to Assess Hippocampus and Frontal-Cortex Pharmacokinetics

Zaril Zakaria and Raj Badhan

1. Whole-body physiologically based pharmacokinetic (PBPK) CNS model

Well-stirred tissues

$$\frac{dC_T}{dt} = \left[\frac{1}{V_T} \times Q_T \times C_{AR}\right] - \left[\frac{1}{V_T} \times Q_T \times \left(\frac{C_T}{f_{up} \times K_p} \times R_b\right)\right] \quad (1)$$

where C_T is the drug concentration of the respective tissues, t is for time, Q_T is the blood flow rate of the tissue, C_{AR} is the arterial drug input, V_T is the volume of the respective tissue compartment, f_{up} is the fraction unbound of drug in plasma, K_p is the tissue-to-plasma partition coefficient and R_b is the blood-to-plasma ratio of the drug.

Brain:

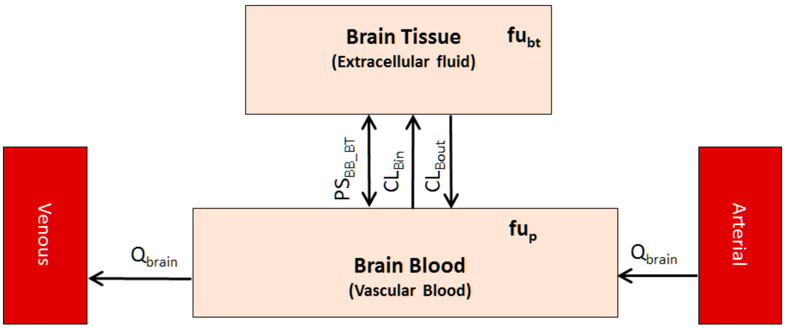

Figure S1. CNS PBPK brain model. Q: blood flow; PS: permeability surface-area; BB: intravascular blood; BT: brain tissue; fu: drug fraction unbound.

$$\frac{dC_{bb}}{dt} = Q_{brain}(C_{arterial} - C_{bb}) + (PS_{BB}C_{bt}fu_b - PS_{BB}C_{bb}fu_p) \quad (2)$$

where C_{bb} is the drug concentration in the brain blood (brain vascular blood), t is for time, Q_{brain} is the blood flow rate to the brain, $C_{arterial}$ is the arterial drug input, fu_p is the fraction unbound of drug in plasma and brain (fu_p), PS is the bidirectional passive permeability-surface area product of BBB and CL_{Bin} and CL_{Bout} are the active clearance into and out of the brain, respectively.

1.1. Compound parameters

Unless otherwise stated, all data contained within the tables below are applied to the rat CNS PBPK model. For a complete description of model parameters, estimation methods (where applied) and references, please see Badhan *et al* (2014) [1].

Table S1. *In-vitro* permeability data.

	Parental cells [a]
	P_{app}

	cm/s (x10[-6])	
	**AB**	**BA**
Benzypenicillin	4.35	3.17
Buspirone	34.25	33.6
Caffeine	27.0	29.7
Carbamazepine	3.45	34.5
Diazepam	37.3	36.8
Midalzolam	34.8	35.5
Phenytoin	20.94	31.7
Sertraline	2.27	1.61
Thiopental	31.2	30.6
Zolpidem	35.6	35.4

[a] Data obtained from [2,3]

Table S2. Model predicted *versus* literature reported *in situ* permeabilities.

	**Permeability Clearance**		**In Situ Permeability**
	**mL/h**		**mL/h**
	**PS_{BB_BT}**	**PS_{BT_BB}**	
Benzypenicillin	4.3	3	0.97 [4]
Buspirone	33.3	32.6	
Caffeine	26.3	28.2	95 ±33.7 [5–8]
Carbamazepine	33.6	33.5	116 [9]
Diazepam	36.3	35.8	351 ±254 [5,9,10]
Midalzolam	33.9	34.5	459 [9]
Phenytoin	25.4	30.8	36.7 ± 21 [8,9,11]
Sertraline	2.2	1.6	129 [12]
Thiopental	30.4	29.8	-
Zolpidem	34.7	34	-

Table S3. Physicochemical parameters used to calculate partition coefficients.

	**Physicochemical parameters** [a]	
	**pKa**	**LogP**
Benzypenicillin	3.55 [b]	2.74 [c]
Buspirone	7.62	1.95 [d]
Caffeine	14	0.92 [e]
Carbamazepine	15.96	2.1
Diazepam	3.4 [c]	2.82 [f]
Midalzolam	6.2	3.89
Phenytoin	8.3 [g]	2.5
Sertraline	9.16 [h]	5.1
Thiopental	7.55 [f]	2.85 [b]
Zolpidem	6.2	1.2

All partition coefficient were subsequently calculated using Rogers and Rowlands mechanistic approaches [13,14].

[a.] Unless otherwise stated, calculated using ChemAxon
[b.] Hansch *et al* (1995) [15]
[c.] Merck Index (2001) [16]
[d.] Ullrich and Rumrich (1992) [17]
[e.] Martin *et al* (1969) [18]
[f.] Sangster (1994) [19]
[g.] McLure *et al* (2000) [20]
[h.] Deak *et al* (2006) [21].

Table S4. Protein binding and metabolic clearance.

	**Protein Binding** [a]			**Metabolic Clearance**
	Plasma	Brain	CSF	Rat [b]
				**CLint, in vivo**
	**fu**plasma	**fu**brain	**fu**CSF	mL/min/kg
Benzypenicillin	0.649	2.26	0.998	na
Buspirone	0.45	0.137	0.996	95.34
Caffeine	0.917	0.697	1	0.70
Carbamazepine	0.385	0.17	0.995	0.37
Diazepam	0.211	0.0426	0.989	0.88
Midalzolam	0.045	0.0431	0.94	75.66
Phenytoin	0.302	0.0967	0.993	0.56
Sertraline	0.0347	0.00038	0.923	158.01 [c]
Thiopental	0.202	0.244	0.988	na
Zolpidem	0.267	0.265	0.992	7.47

[a.] Taken from Kodaira *et al* (2011) [22].

[b.] Unless otherwise indicated, intrinsic *in-vivo* clearance was calculated based on a well-stirred liver model assuming average hepatic blood flow (Q_H, 55 mL/min/kg). Blood clearance and unbound fraction in blood were determined using the blood:plasma ratio (R_b) or by assuming a value of 1 for basic and neutral drugs and 0.55 for acidic drugs.

[c.] Calculated from Ronfield *et al* (1997) [23]

Table S5. Renal Clearance.

**Compound**	**Rat**
	CL_R ml/min/kg
Benzypenicillin	10.22 [a]
Buspirone	na
Caffeine	0.021 [b]
Carbamazepine	na
Diazepam	na
Midalzolam	na
Phenytoin	na
Sertraline	na
Thiopental	na

Zolpidem	na

Renal clearance in rats (CL_R) was calculated based on glomerular filtration rate (GFR) ratio approach as described by Lin (1998) [24]

[a.] Taken from Scavone *et al* (1989 [25], 1997 [26]) and Thompson *et al* (1996) [27]

[b.] Taken from Birkett and Miners (1991) [28].

2. Regional brain PBPK sub-model

Intercranial blood ('brain blood'):

$$\begin{aligned}\frac{dC_{bb}}{dt} = Q_{brain}(C_{art} - C_{bb}) &+ (PS_{FC\_BB}C_{fc}fu_{fc} - PS_{BB\_FC}C_{bb}fu_p) \\ &+ (PS_{BT\_BB}C_{bt}fu_{bt} - PS_{BB\_BT}C_{bb}fu_p) + CL_{Bout}C_{bt}fu_{bt} \\ &- CL_{Bin}C_{bb}fu_p + (PS_{HC\_BB}C_{hc}fu_{hc} - PS_{BB\_HC}C_{bb}fu_p) \\ &+ Q_{CSink}C_{csf}fu_{csf}\end{aligned} \tag{3}$$

where the subscripts *art, bb, bt, csf, hc, fc, p,* and *Csink* represent arterial blood, brain blood, brain tissue, cerebral spinal fluid, hippocampus, frontal cortex, plasma and CSF absorption, respectively; *Q* is blood or CSF flow; *CL* is transporter clearance, respectively; *PS* is the passive permeability-surface area product of BBB, BCSFB, blood brain to hippocampus (and *vice versa*), and blood brain to frontal cortex (and *vice versa*), respectively; *fu* is fraction unbound.

Brain tissue (rest of brain tissue):

$$\begin{aligned}\frac{dC_{bt}}{dt} = (PS_{BB\_BT}C_{bb}fu_p - PS_{BT\_BB}C_{bt}fu_{bt}) &+ CL_{Bin}C_{bb}fu_p - CL_{Bout}C_{bt}fu_{bt} \\ &+ (PS_{FC_{BT}}C_{fc}fu_{fc} - PS_{BT_{FC}}C_{bt}fu_{bt}) - Q_{bulkBT_{CSF}}C_{bt}fu_{bt} \\ &+ (PS_{HC_{BT}}C_{hc}fu_{hc} - PS_{BT_{HC}}C_{bt}fu_{bt})\end{aligned} \tag{4}$$

where Q_{bulkBT_CSF} is the bulk flow from the brain tissue to the CSF.

Frontal cortex:

$$\begin{aligned}\frac{dC_{fc}}{dt} = (PS_{BB\_FC}C_{bb}fu_p - PS_{FC\_BB}C_{fc}fu_{fc}) &- Q_{bulkFC\_CSF}C_{fc}fu_{fc} \\ &+ (PS_{BT\_FC}C_{bt}fu_{bt} - PS_{FC\_BT}C_{fc}fu_{fc})\end{aligned} \tag{5}$$

where Q_{bulkFC_CSF} is the bulk flow from the frontal cortex to the CSF.

Hippocampus:

$$\begin{aligned}\frac{dC_{hc}}{dt} = (PS_{HC\_BT}C_{bb}fu_p - PS_{BT\_HC}C_{hc}fu_{hc}) &- Q_{bulkHC_{CSF}}C_{hc}fu_{hc} \\ &+ (PS_{BB_{HC}}C_{bb}fu_p - PS_{HC_{BB}}C_{hc}fu_{hc})\end{aligned} \tag{6}$$

where Q_{bulkHC_CSF} is the bulk flow from the hippocampus to the CSF.

CSF:

$$\begin{aligned}\frac{dC_{csf}}{dt} = Q_{CSink}C_{csf}fu_{csf} + Q_{bulkBT_{CSF}}C_{bt}fu_{bt} &+ Q_{bulkFC\_CSF}C_{fc}fu_{fc} \\ &- Q_{CSink}C_{csf}fu_{csf}\end{aligned} \tag{7}$$

2.1. Compound specific data

Table S6. Physicochemical parameters for the human regional brain model.

	In-vitro permeability (cm/s x10^{-6})		Permeability clearance (mL/h) [b]		pKa	LogP	fu_{plasma}	fu_{brain}	fu_{CSF}	Rb	$CL_{int,in-vivo}$	CL_R
	**$Papp_{AB}$**	**$Papp_{BA}$**	**PS_{BB_BT}**	**PS_{BT_BB}**							**(mL/min/kg)**	
Carbamazepine	-	-	29818	33818	-	-	-	-	-	-	0.4	na
Phenytoin	-	-	22545	27418	-	-	-	-	-	-	0.47	na
Morphine	1.07[a]	1.06[a]	924	926	8.9[c]	0.89[d]	0.74[e]	0.45[f]	1[g]	1.02[f]	18	na

Data for carbamazepine and phenytoin can be found in supplementary materials section 1.1

[a] Obtained from [29]

[b] PS was calculated as described in Section 2.1 of the main manuscript. PS was subsequently applied to all regional brain compartments through correction for regional tissue weight (See Section 2.2 and assumption 8 of in the manuscript)

c obtained from [30]

d obtained from [31]

e obtained from [32]

f obtained from [33]

g assumed to be 1

References

1. Badhan, R.K.S.; Chenel, M.; Penny, J.I. Development of a physiologically-based pharmacokinetic model of the rat central nervous system. *Pharmaceutics* **2014**, *6*, 97-136.
2. Kalvass, J.C.; Maurer, T.S.; Pollack, G.M. Use of plasma and brain unbound fractions to assess the extent of brain distribution of 34 drugs: Comparison of unbound concentration ratios to in vivo p-glycoprotein efflux ratios. *Drug Metab Dispos* **2007**, *35*, 660-666.
3. Uchida, Y.; Ohtsuki, S.; Kamiie, J.; Terasaki, T. Blood-brain barrier (bbb) pharmacoproteomics: Reconstruction of in vivo brain distribution of 11 p-glycoprotein substrates based on the bbb transporter protein concentration, in vitro intrinsic transport activity, and unbound fraction in plasma and brain in mice. *J Pharmacol Exp Ther* **2011**, *339*, 579-588.
4. Suzuki, H.; Sawada, Y.; Sugiyama, Y.; Iga, T.; Hanano, M. Facilitated transport of benzylpenicillin through the blood-brain barrier in rats. *Journal of pharmacobio-dynamics* **1989**, *12*, 182-185.
5. Tanaka, H.; Mizojiri, K. Drug-protein binding and blood-brain barrier permeability. *J Pharmacol Exp Ther* **1999**, *288*, 912-918.
6. Rapoport, S.I.; Ohno, K.; Pettigrew, K.D. Drug entry into the brain. *Brain Res* **1979**, *172*, 354-359.
7. Takasato, Y.; Rapoport, S.I.; Smith, Q.R. An in situ brain perfusion technique to study cerebrovascular transport in the rat. *The American journal of physiology* **1984**, *247*, H484-493.
8. Liu, X.; Tu, M.; Kelly, R.S.; Chen, C.; Smith, B.J. Development of a computational approach to predict blood-brain barrier permeability. *Drug Metab Dispos* **2004**, *32*, 132-139.
9. Summerfield, S.G.; Read, K.; Begley, D.J.; Obradovic, T.; Hidalgo, I.J.; Coggon, S.; Lewis, A.V.; Porter, R.A.; Jeffrey, P. Central nervous system drug disposition: The relationship between in situ brain permeability and brain free fraction. *J Pharmacol Exp Ther* **2007**, *322*, 205-213.
10. Youdim, K.A.; Qaiser, M.Z.; Begley, D.J.; Rice-Evans, C.A.; Abbott, N.J. Flavonoid permeability across an in situ model of the blood-brain barrier. *Free radical biology & medicine* **2004**, *36*, 592-604.
11. Zhao, R.; Kalvass, J.C.; Pollack, G.M. Assessment of blood-brain barrier permeability using the in situ mouse brain perfusion technique. *Pharmaceutical research* **2009**, *26*, 1657-1664.
12. Summerfield, S.G.; Read, K.; Begley, D.J.; Obradovic, T.; Hidalgo, I.J.; Coggon, S.; Lewis, A.V.; Porter, R.A.; Jeffrey, P. Central nervous system drug disposition: The relationship between in situ brain permeability and brain free fraction. *J Pharmacol Exp Ther* **2007**, *322*, 205-213.
13. Rodgers, T.; Rowland, M. Physiologically based pharmacokinetic modelling 2: Predicting the tissue distribution of acids, very weak bases, neutrals and zwitterions. *J Pharm Sci* **2006**, *95*, 1238-1257.
14. Rodgers, T.; Leahy, D.; Rowland, M. Physiologically based pharmacokinetic modeling 1: Predicting the tissue distribution of moderate-to-strong bases. *J Pharm Sci* **2005**, *94*, 1259-1276.
15. Hansch, C.L., A.; Hoekman, D.. *Exploring qsar: Fundamentals and applications in chemistry and biology*. American Chemical Society: 1995; Vol. 2.
16. O'Neil, M.J. The merck index: An encyclopedia of chemicals, drugs, and biologicals. Merck: Whitehouse Station, N.J., 2001.
17. Ullrich, K.J.; Rumrich, G. Renal contraluminal transport systems for organic anions (paraaminohippurate, pah) and organic cations (n1-methyl-nicotinamide, nmen) do not see the degree of substrate ionization. *Pflugers Archiv: European journal of physiology* **1992**, *421*, 286-288.
18. Martin, A.N. *Physical pharmac,* 2 ed.; Philadelphia, Lea & Febiger,: 1969; Vol. 1.
19. Sangster, J. Octanol-water partition coefficients: Fundamentals and physical chemistry. 1 ed.; Wiley: New York, 1994; Vol. 1.
20. McLure, J.A.; Miners, J.O.; Birkett, D.J. Nonspecific binding of drugs to human liver microsomes. *Br J Clin Pharmacol* **2000**, *49*, 453-461.
21. Deak, K.; Takacs-Novak, K.; Tihanyi, K.; Noszal, B. Physico-chemical profiling of antidepressive sertraline: Solubility, ionisation, lipophilicity. *Medicinal chemistry* **2006**, *2*, 385-389.
22. Kodaira, H.; Kusuhara, H.; Fujita, T.; Ushiki, J.; Fuse, E.; Sugiyama, Y. Quantitative evaluation of the impact of active efflux by p-glycoprotein and breast cancer resistance protein at the blood-brain barrier on the predictability of the unbound concentrations of drugs in the brain using cerebrospinal fluid concentration as a surrogate. *J Pharmacol Exp Ther* **2011**, *339*, 935-944.
23. Ronfeld, R.A.; Tremaine, L.M.; Wilner, K.D. Pharmacokinetics of sertraline and its n-demethyl metabolite in elderly and young male and female volunteers. *Clin Pharmacokinet* **1997**, *32 Suppl 1*, 22-30.

24. Lin, J.H. Applications and limitations of interspecies scaling and in vitro extrapolation in pharmacokinetics. *Drug Metab Dispos* **1998**, *26*, 1202-1212.
25. Scavone, J.M.; Blyden, G.T.; Greenblatt, D.J. Lack of effect of influenza vaccine on the pharmacokinetics of antipyrine, alprazolam, paracetamol (acetaminophen) and lorazepam. *Clin Pharmacokinet* **1989**, *16*, 180-185.
26. Scavone, J.M.; Greenblatt, D.J.; Abernethy, D.R.; Luna, B.G.; Harmatz, J.S.; Shader, R.I. Influence of oral contraceptive use and cigarette smoking, alone and together, on antipyrine pharmacokinetics. *J Clin Pharmacol* **1997**, *37*, 437-441.
27. Thompson, G.A.; St Peter, J.V.; Heise, M.A.; Horowitz, Z.D.; Salyers, G.C.; Charles, T.T.; Brezovic, C.; Russell, D.A.; Skare, J.A.; Powell, J.H. Assessment of doxylamine influence on mixed function oxidase activity upon multiple dose oral administration to normal volunteers. *J Pharm Sci* **1996**, *85*, 1242-1247.
28. Birkett, D.J.; Miners, J.O. Caffeine renal clearance and urine caffeine concentrations during steady state dosing. Implications for monitoring caffeine intake during sports events. *Br J Clin Pharmacol* **1991**, *31*, 405-408.
29. Dale, O.; Nilsen, T.; Olaussen, G.; Tvedt, K.E.; Skorpen, F.; Smidsrød, O.; Vårum, K.M. Transepithelial transport of morphine and mannitol in caco-2 cells: The influence of chitosans of different molecular weights and degrees of acetylation. *Journal of Pharmacy and Pharmacology* **2006**, *58*, 909-915.
30. Roy, S.D.; Flynn, G.L. Solubility behavior of narcotic analgesics in aqueous media: Solubilities and dissociation constants of morphine, fentanyl, and sufentanil. *Pharmaceutical research* **1989**, *6*, 147-151.
31. Illum, L.; Watts, P.; Fisher, A.N.; Hinchcliffe, M.; Norbury, H.; Jabbal-Gill, I.; Nankervis, R.; Davis, S.S. Intranasal delivery of morphine. *Journal of Pharmacology and Experimental Therapeutics* **2002**, *301*, 391-400.
32. Olsen, G.D. Morphine binding to human plasma proteins. *Clinical pharmacology and therapeutics* **1975**, *17*, 31-35.
33. Ball, K.; Bouzom, F.; Scherrmann, J.-M.; Walther, B.; Declèves, X. Development of a physiologically based pharmacokinetic model for the rat central nervous system and determination of an in vitro–in vivo scaling methodology for the blood–brain barrier permeability of two transporter substrates, morphine and oxycodone. *Journal of Pharmaceutical Sciences* **2012**, *101*, 4277-4292.
